# Large-scale acceleration algorithms for a deep convective physical parameterization scheme on GPU

**DOI:** 10.1371/journal.pone.0314606

**Published:** 2024-12-30

**Authors:** Yongfei Wang, Junping Wang, Jiarui Tian, Lin Li, Fangping Ma, Fang Peng, Hu Ke

**Affiliations:** 1 China Energy Dadu River Hydropower Development Co., Ltd., Chengdu, China; 2 Laboratory of Precision Sensing and Control Center, Institute of Automation, Chinese Academy, Beijing, China; 3 School of Information Engineering, China University of Geosciences, Beijing, China; New York University Abu Dhabi, UNITED ARAB EMIRATES

## Abstract

Early warning of geological hazards requires monitoring extreme weather conditions, such as heavy rainfall. Atmospheric circulation models are used for weather forecasting and climate simulation. As a critical physical process in atmospheric circulation models, the Zhang-McFarlane (ZM) deep convective physical parameterization scheme involves computationally intensive calculations that significantly impact the overall operational efficiency of the model. However, many of these calculations are independent and can be computed in parallel. Therefore, this paper proposes a GPU-based acceleration algorithm for the ZM scheme. Based on the computation characteristics of the ZM scheme, we propose its one-demensional and two-demensional acceleration algorithms based on GPU. These algorithms are implemented using CUDA C and compared against a single Kunpeng-920 (Dual Socket) CPU core and the OpenMP version on multi-core CPUs. In the absence of I/O transmission, the proposed algorithm achieves a speedup of 413.6×. Experimental results demonstrate the significant acceleration effect of the proposed algorithms and methods. It is of great significance for the development of deep convective parameterization schemes and their further generalization in climate models. Additionally, we propose a performance optimization method utilizing the CUDA streaming technology to improve data transmission efficiency between CPU and GPU. In the presence of I/O transmission, the proposed algorithm achieves a speedup of 350.1× on A100 GPU.

## 1 Introduction

Affected by Typhoon “Dusurui” (Typhoon No. 2305), Beijing’s Fangshan District experienced prolonged heavy rainfall and intense precipitation during the “23·7” rainstorm, with a cumulative rainfall of 627.1 mm. The excessive floodwater caused significant impact on the river flood control system, exposing obvious shortcomings in China’s defense against excessive floods [1]. In addition to strengthening the investigation of flood hazards, efforts should be made to enhance the capacity for flood forecasting and early warning. Therefore, studying climate models and physical parametrization schemes for rainfall prediction holds significant importance.

CAS-ESM [2] is a component of the Sixth Climate Model Inter-comparison Program (CMIP6) and is used by the Institute of Atmospheric Physics of the Chinese Academy of Sciences for studying the global climate system. It employs intricate physical parameters and atmosphere-ocean interactions to simulate the dynamics of the global climate. CAS-ESM plays a vital role in climate change prediction, analysis of climate variables, and assessing the impact of climate disturbances and policies. The model adopts the Atmospheric Circulation Model Version 4.0 (IAP AGCM4.0) [3] as its atmospheric component, with a focus on accurately simulating the physical process of deep convection. Deep convection, which occurs frequently and influences the exchange of matter and energy in the troposphere, is crucial for maintaining the Earth’s atmospheric system balance. To capture precipitation distribution and changes in different regions, precise simulation calculations are required, making deep convection simulation a critical step in climate research. In recent years, GPUs have gained popularity in meteorology due to their high parallelism and computing capabilities, enabling efficient parallel computing for tasks such as deep convection simulation [4–6].

The ZM process in the CAS-ESM global atmosphere model is very time-consuming, so developing an efficient parallel algorithm is a challenging and meaningful work. After further analyzing the code structure, we found that it is very suitable for parallel development. This paper proposed a GPU-based parallel algorithm that aims to accelerate the CAS-ESM. After implementing the algorithm on the CUDA parallel computing platform, a GPU version of the CAS-ESM, namely GPU-ZM, was developed. The experimental results show that the acceleration algorithm obtained a 350.1× speedup on a single A100 GPU with I/O transfer. Nevertheless, in heterogeneous computing data transfer costs are still significant. Therefore, we propose a data transfer optimization method to reduce transfer time and further improve computing performance. CAS-ESM can achieve a speedup of 413.6× speedup. Our work has realized the parallelization of the overall code from two apects of calculation and data transfer: the parallelism of the CAS-ESM code has been fully exploited, and the data transfer time has been further compressed. Finally, the performance of the code has been evaluated and verified from various aspects.

The numerical calculation and simulation process of deep convection play a crucial role in atmospheric circulation models and require substantial computational resources. To address these challenges, this paper analyzes the numerical calculation process of deep convection while leveraging the NVIDIA GPU parallel architecture CUDA [7]. Based on this analysis, we design a parallel algorithm for the numerical calculation of deep convection using GPU technology. This algorithm aims to accelerate the speed of deep convection calculations and significantly improve computational efficiency.

This article makes the following key contributions:

- By considering the computation characteristics of the ZM deep convection scheme, its GPU-based one-demensional (1D) parallel acceleration algorithm in the horizontal dimension is proposed. Additionally, we also propose its two-demensional (2D) parallel acceleration algorithm which can achieve higher computational efficiency.
- A performance optimization method for data transmission between CPU and GPU is proposed. It utilizes CUDA streaming technology and can improve data transmission efficiency of the ZM scheme.

The rest of this paper is structured as follows. Sec. 2 provides an overview of related work on accelerating physical parameterization schemes using GPUs. Sec. 3 outlines the model and code structure of the ZM scheme. Sec. 4 introduces the GPU-based acceleration algorithm and describes its implementation. Sec. 5 presents the evaluation of the GPU-ZM performance. Sec. 6 summarizes the findings of the study and provides recommendations for future research.

## 2 Related work

Modern GPUs feature highly parallel microprocessors that offer high-performance capabilities for parallel computing in science and engineering. Consequently, GPUs have emerged as robust alternatives to conventional microprocessors in high-performance computing (HPC) systems [8, 9]. Extensive research and experimental investigations have been carried out on GPU-based acceleration algorithms for cloud microphysical parameterization methods across different models and platforms, leading to significant advancements in acceleration outcomes. Mielikainen et al. utilized a single GPU to accelerate the Weather Research and Forecasting (WRF) model with the single-moment Type 5 (WSM5) scheme, resulting in a 389-fold speedup without I/O (input/output) transmission [10]. When using four GPUs, the WRF WSM5 model achieved performance improvements of 357× and 1556× with and without I/O transmission, respectively. In the same year, Mielikainen et al. proposed a GPU-based implementation of the Kessler microphysics scheme [11]. Similarly, the WRF two-moment class 6 (WDM6) microphysics scheme, also developed by Mielikainen et al., achieved speedups of 150× and 206× with and without I/O transmission, respectively [12]. Leutwyler et al. implemented a GPU version of the convection-resolved COSMO model within a climate model and successfully integrated it into a multi-node heterogeneous atmosphere model. They demonstrated the applicability of this approach by conducting a three-month simulation [13]. Cao et al. implemented the AGCM-3DLF, a highly scalable 3D atmospheric circulation model based on the leap format. Experimental results on different platforms showed that the model exhibited excellent efficiency and scalability, achieving a speed of 11.1 simulated years/day (SYPD) at a high resolution of 25 km on the CAS-Xiandao-1 supercomputer [14]. Huang et al. presented a parallel design of a GPU-based WSM6 scheme, which achieved a 216× speedup using a single NVIDIA K40 GPU compared to the CPU portion running on a single core [15]. Kim et al. proposed a scheme to accelerate the microphysics of the WSM6 scheme in the cross-scale prediction model using OpenACC instructions. They verified the performance of the code on the NVIDIA GPU Tesla V100 and achieved a 5.71× speedup without I/O transfer when the entire model was ported to the GPU [16]. Zhenzhen Wang et al. developed two algorithms using GPU technology to accelerate the shortwave radiation scheme (RRTMG_SW) of RRTMG. They also proposed an optimization method for data transmission between the host and the device. By implementing these algorithms using CUDA Fortran and CUDA C, two GPU versions of RRTMG_SW, CF-RRTMG_SW, and CC-RRTMG_SW, were created. The solution running on a single NVIDIA GeForce Titan V achieved a 38.88× faster performance than the baseline running on a single Intel Xeon E5-2680 CPU core [17]. Li et al. proposed a three-dimensional acceleration algorithm for CC-RRTMG_SW and studied optimization methods such as decoupling data dependence, optimized memory access, and I/O optimization. As a result, CC-RRTMG_SW was developed [18]. Hong et al. proposed GPU-based one-dimensional (1D) and two-dimensional (2D) parallel acceleration algorithms for the CAM5 Cloud Microphysics Scheme (CAM5 CMS) by analyzing its parallelism in different dimensions. The 2D parallel algorithm took advantage of finer-grained parallelism and an optimization method for data transfer between the CPU and GPU was proposed to further enhance overall performance [19].

Initially, GPUs were primarily utilized for image rendering purposes. However, the introduction of NVIDIA’s CUDA parallel computing platform has effectively harnessed the immense computational capabilities of GPUs, resulting in significant acceleration in computationally intensive tasks involving large-scale parallel data processing [9]. On the other hand, CPUs are well-suited for handling control tasks involving complex logic, leading to the development of heterogeneous CPU/GPU parallel computing systems. The CUDA platform, with its maturity and stability, has become a preferred choice for parallel programming in numerous scientific research and experimental endeavors. Furthermore, a multitude of proven applications have been developed using CUDA C. In summary, GPUs offer a suitable solution for accelerating the physical parameterization of climate system models. Currently, there is a lack of research focusing on the utilization of GPUs to accelerate deep convective physical processes. Consequently, this paper opts to implement the ZM deep convection model algorithm in CUDA C and explore its one-dimensional and two-dimensional acceleration algorithms based on GPU architecture, leveraging the substantial computational power offered by GPUs.

## 3 Model description

### 3.1 ZM scheme

Deep convection is a convective system with a vertical thickness of the same order as the homogeneous atmospheric height, and the performance of deep convection in GCMs has an important impact on the simulation of global climate and its changes due to the strong interaction between deep convection and large-scale circulation. In this paper, we will use the ZM scheme to simulate the annual precipitation cycle mode, which will be used to simulate the change and distribution of precipitation. The calculation process of the computational model is as follows [20].

Convective effective potential energy *CAPE* is defined as formula (1).

$$\begin{array}{c} \begin{array}{c} CAPE=\int_{P_{t}}^{P_{b}}R_{d}\left(T_{vp}-T_{ve}\right)d\;{In}_{p} \end{array} \end{array}$$ (1)

where *Tvp* is the virtual temperature of the gas block, *Tve* is the virtual temperature of the environment, *Rd* is the dry gas constant, *Pb* is the air pressure at the beginning of the gas block, and *Pt* is the ground air pressure.

The changes caused by *CAPE* during convection are:

$$\begin{array}{c} \begin{array}{cc} \frac{\partial A}{\partial t} & =\frac{\partial }{\partial t}\{\int_{P_{t}}^{P_{b}}R_{d}\left(T_{vp}-T_{ve}\right)d\;{In}_{p}\}\\ \; & =\int_{P_{t}}^{P_{b}}R_{d}\left(\frac{\partial T_{vp}}{\partial t}-\frac{\partial T_{ve}}{\partial t}\right)d\;{In}_{p}-R_{d}{\left(T_{vp}-T_{ve}\right)}_{p=pt}\frac{\partial T_{t}}{\partial t} \end{array} \end{array}$$ (2)

While the theoretical definition states that *Tvp = Tve*, in practical applications, the finite difference method used for the time derivative occasionally results in a small non-zero value for the last term. This occurs when there are temporal changes in the neutral buoyancy level. However, when calculating the change in *CAPE*, we disregard this term. Thus, the equation can be simplified as follows:

$$\begin{array}{c} \begin{array}{c} \frac{\partial A}{\partial t}={\left(\frac{\partial A_{p}}{\partial t}\right)}_{cu}+{\left(\frac{\partial A_{p}}{\partial t}\right)}_{ls}+{\left(\frac{\partial A_{e}}{\partial t}\right)}_{cu}+{\left(\frac{\partial A_{e}}{\partial t}\right)}_{ls} \end{array} \end{array}$$ (3)

Four of these variations are obtained by the follows:

$$\begin{array}{c} \begin{array}{c} {\left(\frac{\partial A_{p}}{\partial t}\right)}_{cu}=R_{d}\int_{P_{t}}^{P_{b}}{\left(\frac{\partial T_{vp}}{\partial t}\right)}_{cu}d\;{In}_{p} \end{array} \end{array}$$ (4)

$$\begin{array}{c} \begin{array}{c} {\left(\frac{\partial A_{p}}{\partial t}\right)}_{ls}={-R}_{d}\int_{P_{t}}^{P_{b}}{\left(\frac{\partial T_{vp}}{\partial t}\right)}_{ls}d\;{In}_{p} \end{array} \end{array}$$ (5)

$$\begin{array}{c} \begin{array}{c} {\left(\frac{\partial A_{e}}{\partial t}\right)}_{cu}=R_{d}\int_{P_{t}}^{P_{b}}{\left(\frac{\partial T_{ve}}{\partial t}\right)}_{cu}d\;{In}_{p} \end{array} \end{array}$$ (6)

$$\begin{array}{c} \begin{array}{c} {\left(\frac{\partial A_{e}}{\partial t}\right)}_{ls}={-R}_{d}\int_{P_{t}}^{P_{b}}{\left(\frac{\partial T_{ve}}{\partial t}\right)}_{ls}d\;{In}_{p} \end{array} \end{array}$$ (7)

And the cloud base mass flux *Mb* is:

$$\begin{array}{c} \begin{array}{c} M_{b}=\frac{1}{k}max\{-\int_{P_{t}}^{P_{b}}{\left(\frac{\partial T_{ve}}{\partial t}\right)}_{ls}d\;\text{ln}\;p,0\} \end{array} \end{array}$$ (8)

The *k* in Eq (9) is:

$$\begin{array}{c} \begin{array}{c} k=\int_{P_{t}}^{P_{b}}\left(1+0.608q\right)\left[-\eta \frac{\partial S}{\partial p}\right]+0.608T\left[-Sp+qs-q\right]dInp \end{array} \end{array}$$ (9)

In Eq (10), *S* is the clean energy, *q* is the specific moisture, and *qs* is the saturation specific moisture. From this, the rate of change in humidity can be found:

$$\begin{array}{c} \begin{array}{c} {\left(\frac{\partial q}{\partial p}\right)}_{cu}=M_{b}\left[-\eta \frac{\partial q}{\partial p}+\delta \left(q_{s}-q\right)\right] \end{array} \end{array}$$ (10)

### 3.2 Algorithm structure

**Fig 1:**
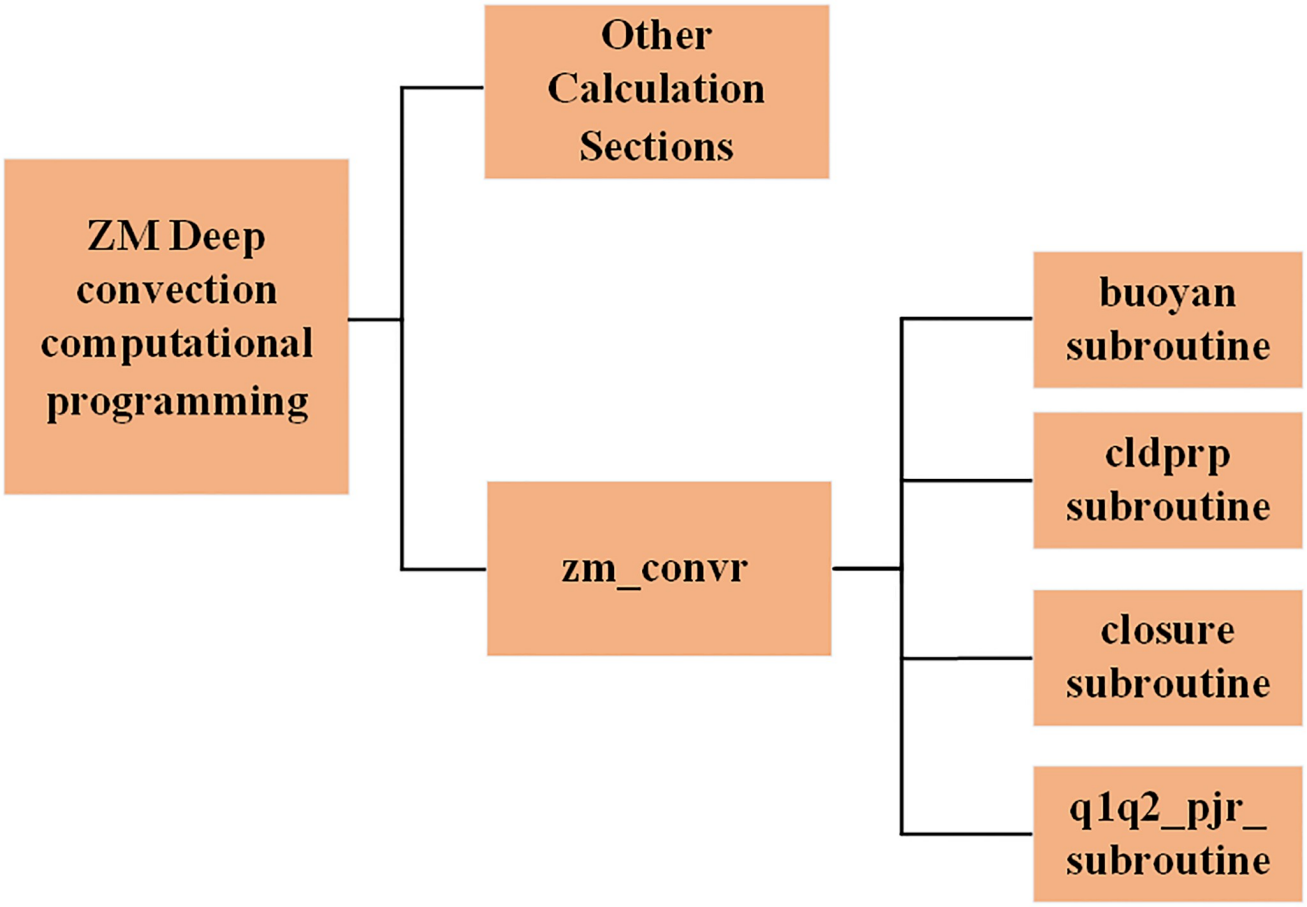
ZM deep convection code structure.

The algorithm structure of the ZM scheme is depicted in Fig 1. The core computation of the algorithm resides in the *zm_convr* subroutine. When designing the parallel algorithm, particular attention is given to the *zm_convr* subroutine, as well as the four functions: *buoyan*, *cldprp*, *closure*, and *q1q2_pjr*, which are invoked during the calculation process. These four functions will be used as kernel functions. Additionally, the parallelizable computations within the *zm_convr* subroutine will be identified and separated to facilitate the independent design of the parallel algorithm. The ZM scheme utilizes the *zm_convr* subroutine for iterative calculations.

In this algorithm, the *zm_convr* subroutine first initializes the array values and related parameters required for the calculation. It then calls the *buoyan*, *cldprp*, *closure*, and *q1q2_pjr* functions to compute the relevant values. The computation results are returned to the *zm_convr* subroutine, which further organizes the results to obtain the necessary data for deep convection calculations.

## 4 GPU-based acceleration algorithm

### 4.1 Algorithm analysis

Similar to the real Earth, CAM-ESM describes the atmosphere in a three-dimensional form. The *x*-axis, *y*-axis, and *z*-axis represent the longitude, latitude, and model levels (vertical direction) of the Earth, respectively. The computations in the longitude and latitude dimensions (horizontal direction) of CAS-ESM are usually independent, and there is partial independence in the vertical direction, so 1D and 2D parallelization can be achieved in these two dimensions. In CAS-ESM, the longitude and latitude dimensions are merged as the first dimension of the parameter arrays, and the vertical direction is the second dimension. Data parallelism is mainly achieved through the thread and block indices of the CUDA architecture, which provide a unique global index for each thread, allowing multiple threads to execute the same computation on different data, which is the single instruction, multiple data (SIMD) mode [19]. In the experiments, the ZM is with a horizontal resolution of 0.7°×1.4°, resulting in a grid of 256×256 = 65536 horizontal points, and it also includes 51 model layers in the vertical direction. To verify the authenticity of the experiments, we also used three scales of 128×128 = 16384, 256×128 = 35768 for verification, and the specific experimental content is presented in Sec. 5.

Since further testing found that the effect was best with a block size of 65536, that value is used as an example here. During single-GPU acceleration, 65,536 horizontal grid points are computed at each time step. The model is typically computed independently in latitude and longitude (horizontal dimensions) and partially independently in the vertical axis, facilitating parallelism in both 1D and 2D directions. When implementing the ZM deep convection physical model, the latitude and longitude dimensions are consolidated into the first dimension of the parameter array, while the vertical direction remains as the second dimension. Data parallelism is achieved by utilizing thread and block indices in the CUDA architecture. Each thread is assigned a distinct global index, enabling multiple threads to execute the same computation on different data, following the Single Instruction Multiple Data (SIMD) paradigm.

Within the program structure, the variable ncol signifies the extent of the horizontal dimension, dictating the overall count of grid points. For instance, if ncol is assigned a value of 2048, the 65,536 grid points will be partitioned into 32 blocks, each containing 2048 points. Consequently, during each time step, the GPU will execute a total of 65,536 calculations over 32 iterations.

**Fig 2:**
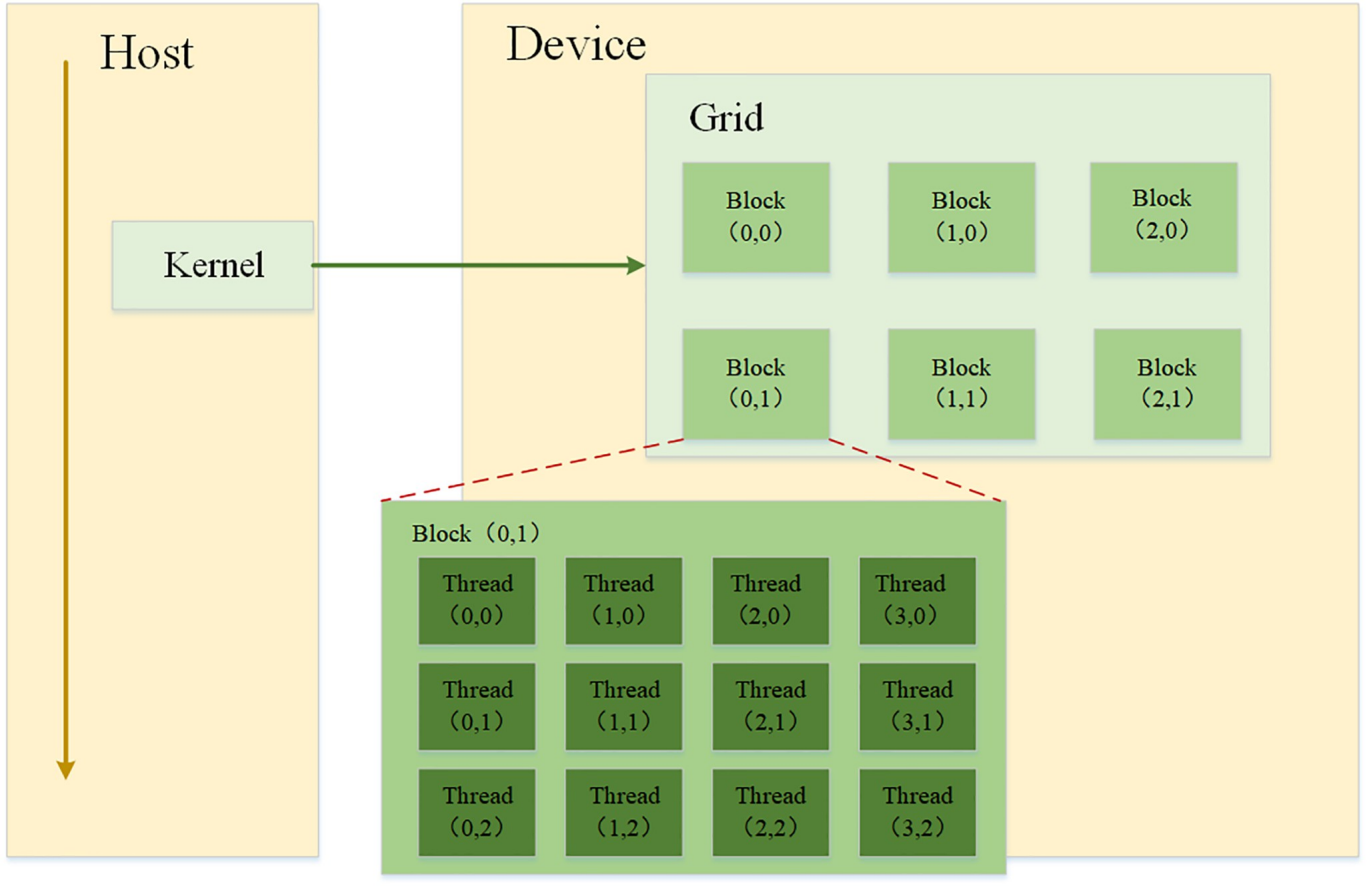
CUDA execution model.

As depicted in Fig 2, CUDA’s execution model comprises a three-tier hierarchy of threads. Each core operates on a grid, which encompasses multiple blocks, and each block consists of numerous threads. All threads within the same grid share a common global memory space. Intra-block collaboration is facilitated through synchronization and shared memory, while inter-block collaboration is not possible. Threads within the grid are identified using two scalar coordinates: blockIdx (representing the block’s ID number within the grid) and threadIdx (representing the thread’s ID number within the block). Within the CUDA programming model, kernel functions are invoked using the “kernel” ⋘ *grid, block* ⋙ statement, where “grid” represents the number of thread blocks and “block” indicates the number of threads within each block. By adjusting the execution configuration of the kernel, different computing performance can be achieved, as explained in Sec. 5.

In the context of CPU/GPU heterogeneous computing, we encapsulate the original program into a callable function and refactor each loop into separate subfunctions that can be invoked as kernels. These kernel functions are designed to be executed on the device while being called from the host. By leveraging this heterogeneous computing paradigm, we aim to enhance the computing performance of ZM. The execution processes for the GPU-ZM algorithm can be summarized as follows:

(1) Declare and allocate memory on the CPU for arrays and variables involved in computations on the host. Simultaneously, define and allocate memory on the GPU for data that requires computation on the device, encompassing inputs, outputs, intermediate arrays, and variables.
(2) Initialize the data, including input and output arrays, as well as other parameters.
(3) Copy the initialized data to the global memory of the GPU.
(4) Determine the suitable thread block and grid dimensions, and invoke the kernel to execute the raster, grid, and other functions for computing the microphysics process.
(5) Retrieve the computed results from the GPU and transfer them to the CPU.
(6) Output the results to free the memory on both the GPU and CPU.

In the deep convection physical model, the core computational program *zm_convr* serves as the primary driver for the ZM convection scheme. Its calculations are primarily based on the mass flux *Closure* algorithm for adjusting deep convection. The algorithm’s flow is outlined in Algorithm 1. The *zm_convr* program comprises four computational subroutines. The *buoyan* subroutine is responsible for describing the algorithm used in the operation and includes any relevant external reference data. It calculates the convection top of the Convective Available Potential Energy (CAPE), lifting the condensation level and determining *buoyan*. The *buoyan* computation program predominantly involves calculations in the horizontal direction, which are independent. Therefore, when designing the parallel algorithm, we primarily divide the calculation part into two-dimensional calculations. The *cldprp* subroutine is mainly employed to calculate the properties of cumulus clouds. The program performs independent calculations in both the horizontal and vertical dimensions. As a result, we divide the calculation in the horizontal dimension into two dimensions, while the calculation in the vertical dimension is performed in one dimension. The *closure* subroutine aims to restrict the mass flux of cumulus clouds to the theoretical upper limit. Given that both the *closure* subroutine and the *cldprp* program involve cumulus cloud calculations, the algorithm design approach for this program is similar to that of *cldprp*, with the horizontal dimension calculation being divided into two dimensions. The *q1q2_pjr_* subroutine calculates the temperature and humidity changes caused by convection. The algorithm primarily performs independent calculations in both the horizontal and vertical directions. Consequently, we also design the program with a focus on 2D division.

To summarize, the parallel algorithm design for the deep convection physical model involves dividing the calculations in the horizontal dimension into 2D, while the calculations in the vertical dimension are divided into one or two dimensions, depending on the specific subroutine.

**Algorithm 1** Deep Convective Adjustment Algorithm

**Function**: ZM_convr

01: **Data Initialization**

02: **Function** *convr()*:

03:  **CUDADataInit**

04:  **DataMemcpy: Host to Device**

   //Compute the local pressure (in millibars) and height (in meters) for both interface and mid-layer locations.

05:  **call** *zm_convr_kernel1*

06:  **if** *cam physpkg is cam3* **then**

07:    **call** *buoyan*

08:  **end**

   // Obtain gathered arrays necessary for ensuing calculations.

09:  **call** *zm_convr_kernel2*

   // Obtain cloud properties.

10:  **call** *cldprp*

   // Convert the detrainment units from “*1/m*” to “*1/mb*”.

11:  **call** *zm_convr_kernel3*

   // Convert the detrainment from units of “*1/m*” to “*1/mb*”.

12:  **call** *closure*

   // Constrain the cloud base mass flux to its theoretical upper limit.

13:  **call** *zm_convr_kernel4*

   // Calculate the variations in temperature and moisture resulting from convection.

14:  **call** *qlq2_pjr_*

   // Gather back temperature and mizing ratio.

15:  **call** *zm_convr_kernel5*

16:  **DataMemcpy: Device to Host**

17:  **DataFree**

18: **return**

### 4.2 Acceleration algorithms

#### 4.2.1 1D acceleration algorithm

**Fig 3:**
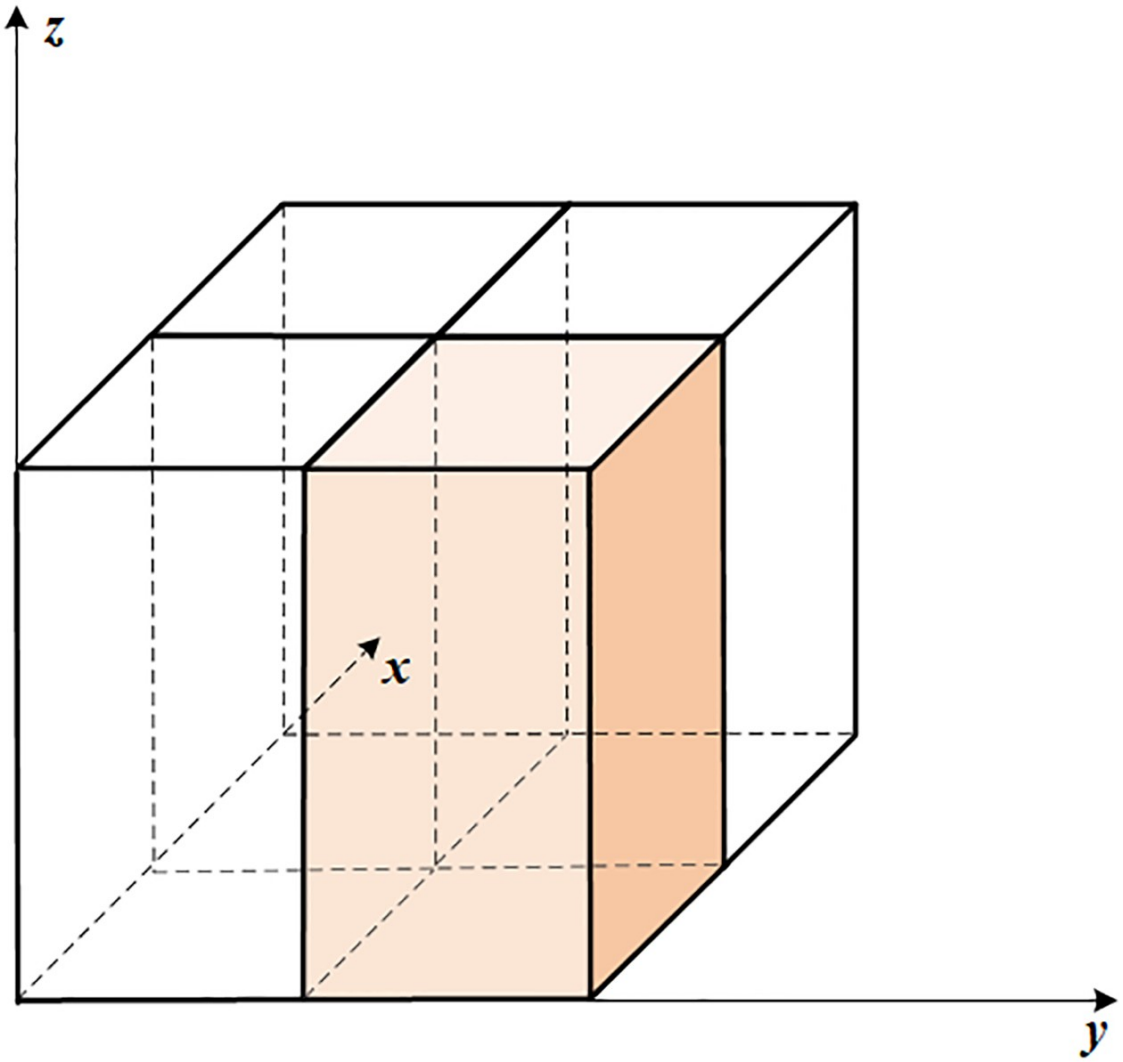
One-dimensional partition space scheme.

The ZM scheme utilizes a three-dimensional (3D) grid structure consisting of horizontal and vertical layers, as depicted in Fig 3. During the calculation process, the 3D spatial mesh is mapped onto a 2D array. Due to the horizontal direction’s independence in the ZM, a 1D parallel strategy is employed for regional decomposition in the horizontal direction (latitude and longitude dimensions). This means that each CUDA parallel thread is assigned a horizontal “bar” workload, as illustrated in Fig 3. The size of the 1D of the ncol variable in the code indicates the number of “bars” participating in parallelism. Considering the order and dependencies of the code structure, the parallel code is organized into four core functions. These core functions are executed sequentially, while the three kernel functions are named *buoyan*, *cldprp*, *closure*, and *q1q2_pjr_*.

#### 4.2.2 2D acceleration algorithm

**Fig 4:**
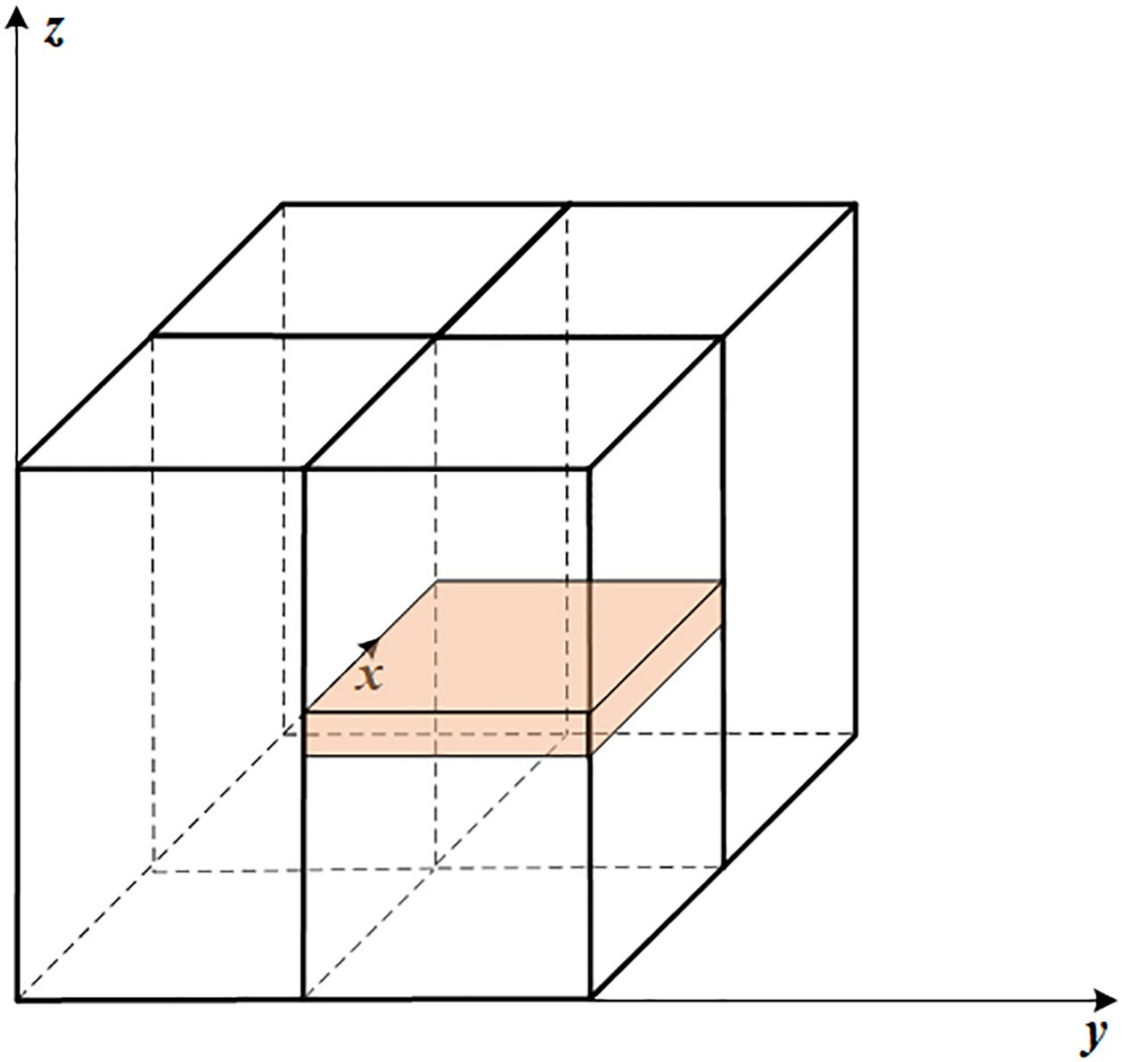
Two-dimensional partition space scheme.

In order to achieve a higher level of parallelism on GPUs, make full use of GPU resources, open up more parallel threads, and introduce 2D parallel acceleration algorithms for more fine-grained parallelism. The approach employed in the ZM is partially autonomous in the vertical direction. To enhance the computational efficiency of the GPU, we made additional modifications to the original code, enabling parallelization in both the horizontal and vertical dimensions. During the 2D region decomposition, each CUDA parallel thread is allocated data from a horizontal grid,as shown in Fig 4.

Further analysis of the code shows that in addition to the four subfunctions, the *zm_convr* function can be further decoupled into five subfunctions *zm_convr1*, *zm_convr2*, *zm_convr3*, *zm_convr4*, and *zm_convr5*. Because *zm_convr2* has a dependency in the vertical direction, only 1D acceleration occurs in the horizontal dimension, and the other functions can accelerate in the horizontal and vertical dimensions. Algorithm 2 shows the implementation of buoyan’s 2D acceleration kernel function, and the implementation of the rest of the 2D acceleration algorithms is similar, so we will not repeat it.

In the case of 1D acceleration, each thread possesses a private 1D temporary array, meaning that each horizontal bar has its own temporary array. This array is stored in registers or local memory. In contrast, in the 2D acceleration, as both the horizontal and vertical dimensions are parallelized, the benefits of optimized memory access through registers are achieved.

**Algorithm 2** Buoyan

01: **Function**: *buoyan()*:

02:  *i* = blockDim.*y* * blockIdx.*y* + threadIdx.*y*;

03:  *k* = blockDim.*x* * blockIdx.*x* + threadIdx.*x*;

04:  Initialize *tv* and *buoyan* for output;

05:  **if** (*k* ≥ 0 and *k* < pver) and (*i* ≥ 0 and *i* < ncol) **then**

   // Reset max moist static energy level (hmn)

06:   calculate hmn

   // calculate lifting condensation level (LCL)

07:   calculate LCL;

08:  **end**

   // Initialize the properties of the parcel within the sub-cloud layer below LCL.

09:  **if** (*i* ≥ 0 and *i* < ncol) **then**

10:   calculate *tv, tp, tpv, buoy*;

11:  **end**

   // Define the properties of the parcel at LCL, specifically at the level immediately above the parcel’s lifting point (PL).

12:  **main buoyancy calculation**;

13: **return**

### 4.3 Data transmission optimization

**Fig 5:**
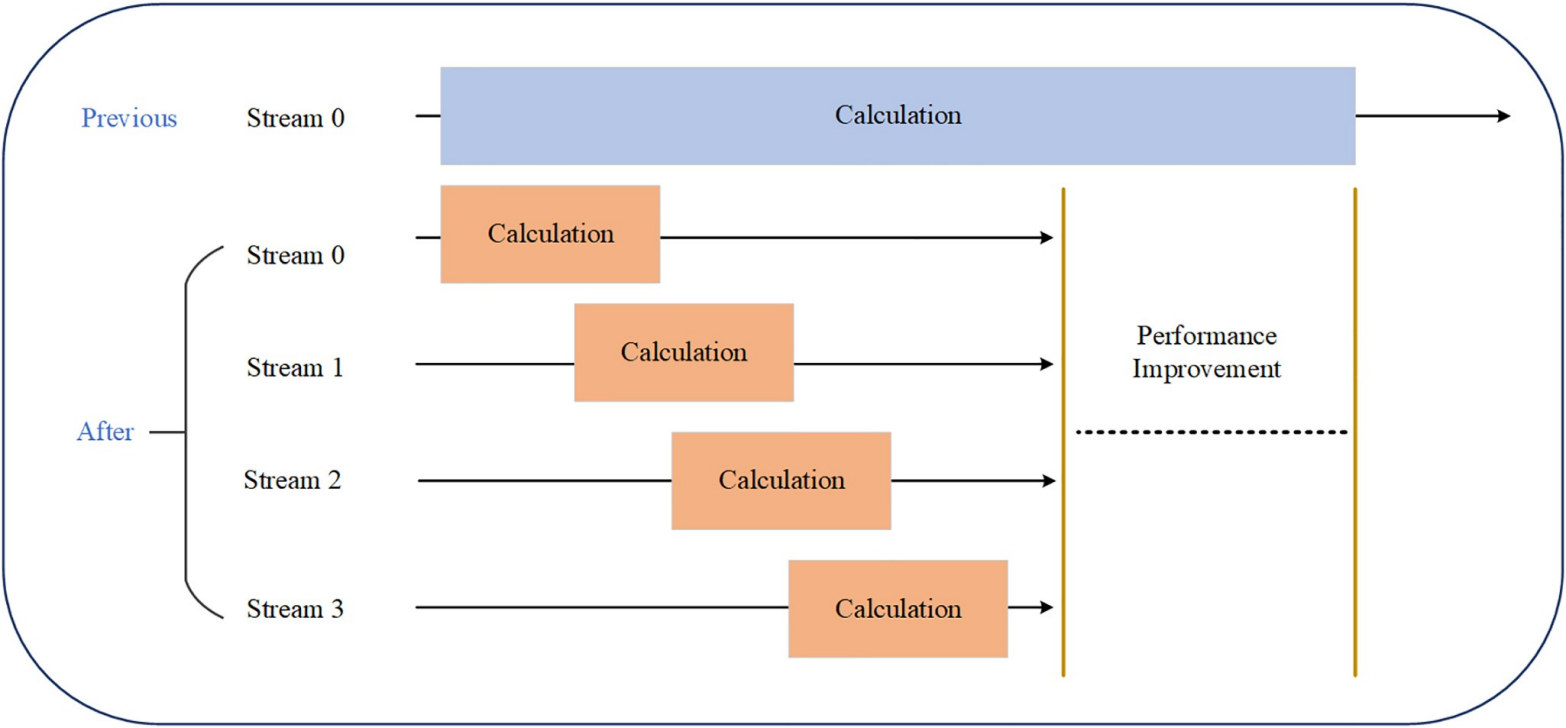
Performance improvement comparison of using stream processing technology.

To enhance the efficiency of data transfer between the CPU and GPU and enhance the overall performance of the algorithm, various techniques can be utilized. One effective solution is to optimize data transfer and utilize CUDA streams for asynchronous transfer. The impact of utilizing CUDA streams on performance can be observed in Fig 5.

Since GPUs consist of multiple Streaming Multiprocessors (SMs), concurrent execution of computation and memory access is possible. By utilizing multiple CUDA streams, performance can be enhanced. CUDA streams have no dependencies between each other, allowing computation and memory access to be allocated to different streams. For example, the current data calculation and the access to data required for the next calculation can be executed concurrently. After the CPU initiates core functions and passes them to the GPU, the CPU can perform other operations instead of waiting for the GPU task to complete. Without using CUDA streams, the CPU would have to wait for the GPU task to finish before proceeding with other operations. By utilizing CUDA streams, the CPU and GPU can execute in parallel, resulting in significant time savings.

A CUDA stream can be considered as a pipeline. Different pipelines can execute concurrently, but operations within the same stream must be executed sequentially. When employing CUDA streams, there are two approaches to consider. One approach is to schedule the same kernel function to run on different streams. This method involves dividing the array data into different parts, transferring each part to a separate stream for use by the kernel functions, and then passing back the computed result data. This method requires the data calculation of each part of the algorithm to be independent. The other approach is to assign different kernel functions that can be computed independently and execute them in different streams. This enables concurrent execution through overlapping data transfers and function operations.

## 5 Results and discussion

### 5.1 Experimental configuration

In the experiments in this paper, the parallel code of ZM deep convection is run on the A100 cluster of the N32 branch of the Beijing Super Cloud Computing Center. Table 1 lists their detailed configurations, including OS size, memory size, memory bandwidth, and CUDA version. It is worth noting that the test cases in the experiment include the ZM serial code running on a single Kunpeng-920 (Dual Socket) CPU core in the A100 cluster, the OpenMP code running on four CPU cores, and the CUDA code (https://github.com/tianjr0423/CUDA-version) using a single GPU. This is based on the GPU-ZM running on a single GPU node in the cluster. Different execution parameters of kernel functions and scale sizes will lead to different code running times and different acceleration effects. Therefore, we have found the optimal configuration parameters through continuous practice and analysis. Based on this, we have also optimized the transmission time using CUDA stream and achieved good results.

**Table 1:** Experimental results for layered mixed-precision training.

Specification of GPU	GPU	CUDA Cores	Standard Memory	Memory Band-width	CUDA Version
A100 cluster	NVIDIA A100	6912	40GB	1555GB/s	12.3

### 5.2 Runtime comparisons under different data scales

In this section, we compare the runtime (without considering I/O transmission) of the single-CPU serial code, the four-core CPU OpenMP parallel code, and the one-dimensional and two-dimensional parallel code based on GPU. We found that there is always a significant performance gap between the single-CPU serial code, the four-core CPU OpenMP parallel code, and the GPU-based one-dimensional and two-dimensional parallel code, regardless of the data scale of 128×128, 128×256 or 256×256.

**Fig 6:**
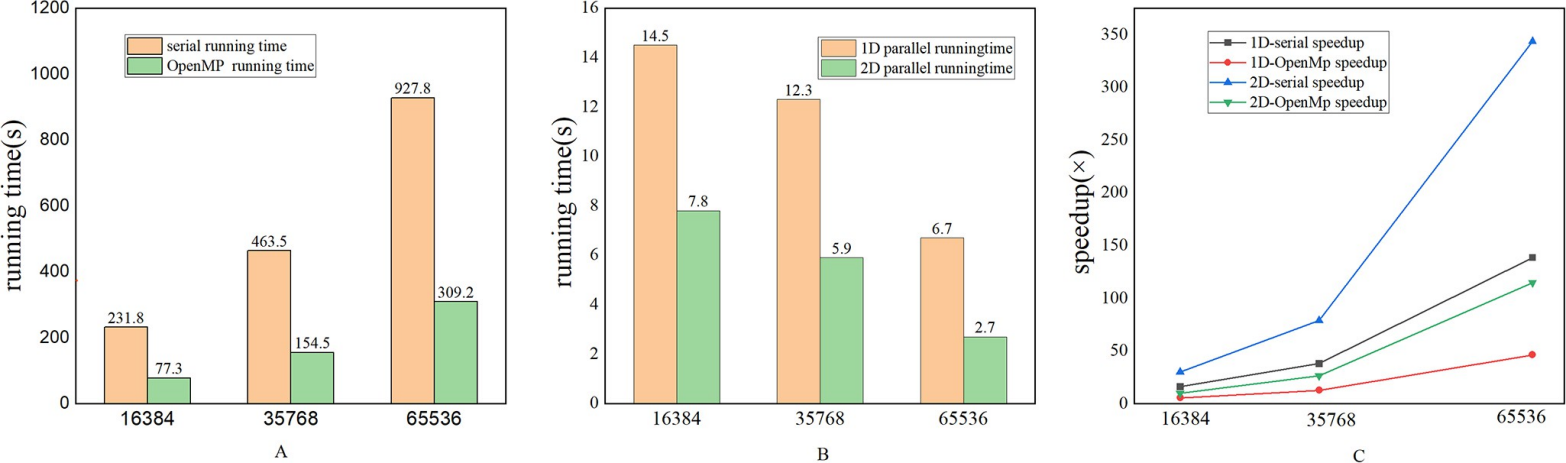
Time measurements for serial, multi-CPU, and GPU parallel executions under different scales.

Specifically, at the smallest scale of 128×128, the runtime of the GPU parallel code is already significantly shorter than the CPU serial and OpenMP parallel codes. As the data scale increases, this gap becomes larger and larger. As shown in Fig 6, when the computing scale is 256×256, the 2D parallel code achieved a speedup of 343.6× compared to the serial code. This fully demonstrates the advantage of the powerful parallel computing capability of GPUs in large-scale data processing. While the CPU OpenMP parallel code also has a certain acceleration compared to the serial code, it is limited by factors such as the number of cores and memory bandwidth, and the performance improvement is relatively small. Therefore, our CUDA parallel rewriting is meaningful.

### 5.3 Effect of ncol

**Fig 7:**
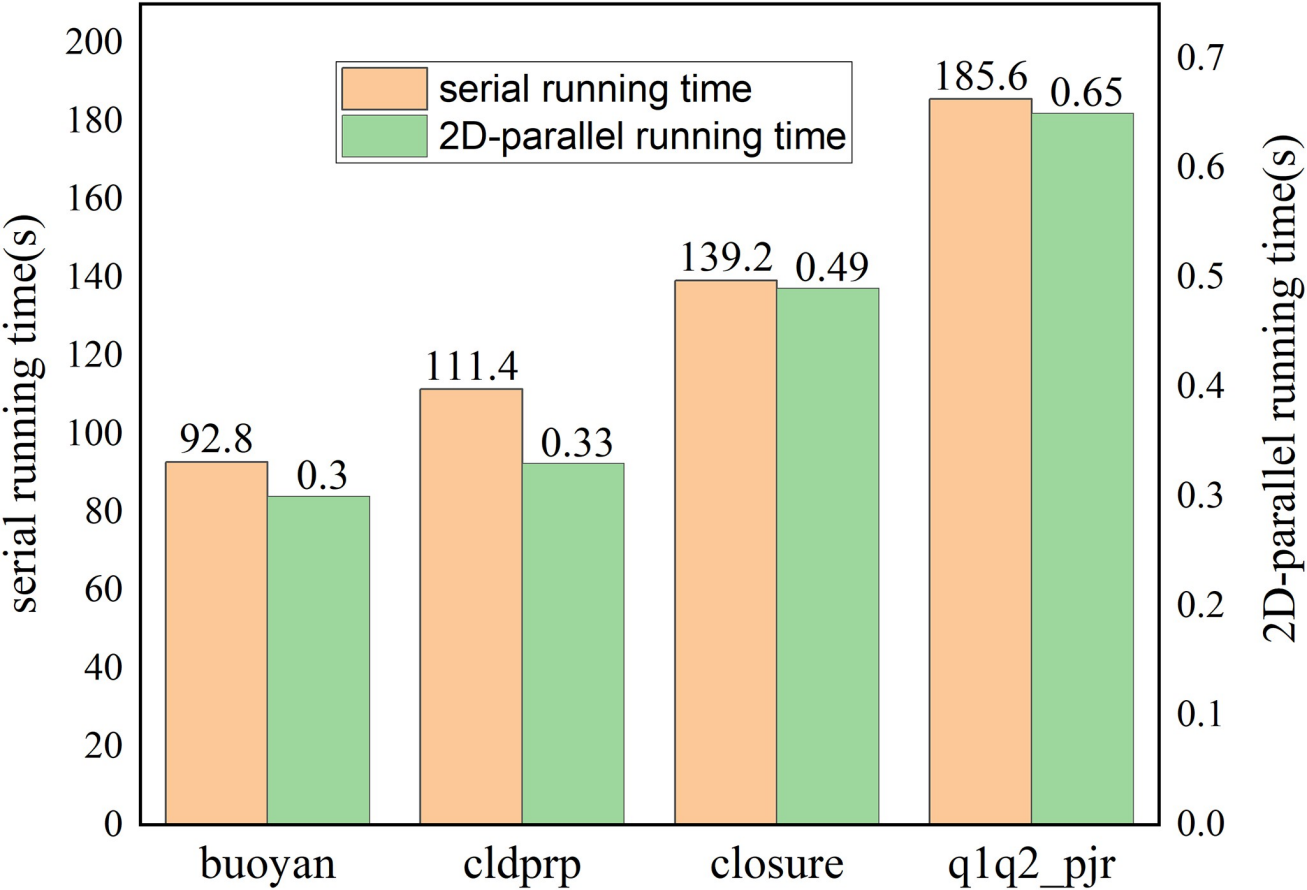
The comparison of serial and parallel runtime (s) for the four sub-functions.

We test the one-dimensional partition scheme and find that the speedup is far worse than the two-dimensional scheme, so the experimental part mainly studies the acceleration effect of two-dimensional parallelism. As the vertical dimension comprises only 51 cells, the focus is primarily on investigating the impact of varying the size of the horizontally parallelizable grid. In the code, the size of the parallel horizontal column grid is denoted by ncol, which is adjusted to calculate the horizontal grid points simultaneously by modifying its value. By increasing the value of ncol, the number of threads in the parallel horizontal dimension is augmented, resulting in a reduction in the number of core functions invoked on the CPU side. This leads to a significant improvement in the performance of the ZM code. In the A100 GPU experiment, the value of ncol was varied from 512 to 65536. When the ZM code employs the maximum value of ncol at 65536, it necessitates invoking the core function 512 times on the CPU. In this experiment, the thread block size is fixed at 256 without considering I/O transmission, and the one-dimensional parallel acceleration code running time change is selected to illustrate the impact of ncol. Fig 7 illustrates the runtime and speedup ratio of the ZM one-dimensional parallel algorithm as a function of the ncol value. Based on the observations and results, the following conclusions can be drawn:

(1) Increasing the value of ncol reduces the running time of the *zm_convr* function and improves the speedup ratio. Doubling the ncol value increases the parallelism rate of the GPU-ZM program, so that the interval between runs is also about halved. However, when the ncol value exceeds 16384, the memory overhead also increases, so the speedup ratio does not increase significantly. When the ncol value exceeds 32768, the performance advantages of the A100 cluster are fully exerted, and the acceleration ratio is greatly improved.
(2) When the ncol value is 65536, the 1D GPU-ZM achieves the best performance. The acceleration ratio of 364× was obtained, and the run time was compressed from 982.8s to 2.7s.

### 5.4 Effect of block sizes

In the context of parallelism, numerous factors influence the runtime of a GPU-accelerated version. Within the CUDA framework, the size of the thread block is one of these influential factors. Specifically, the quantity and dimensions of threads within an execution block indirectly impact the computational time. Hence, this experimental section aims to investigate the optimal configuration of thread blocks. Typically, a group of threads, referred to as a thread warp, constitutes the fundamental execution unit within a Streaming Multiprocessor (SM). On the other hand, a thread block serves as the fundamental unit of activation. From a hardware standpoint, a thread block is composed of a set of one-dimensional thread warps, with each warp consisting of 32 consecutive threads. Within a thread warp, all threads are executed in a single-instruction multithreaded (SIMT) fashion. Consequently, the block size should be selected as a multiple of 32.

**Fig 8:**
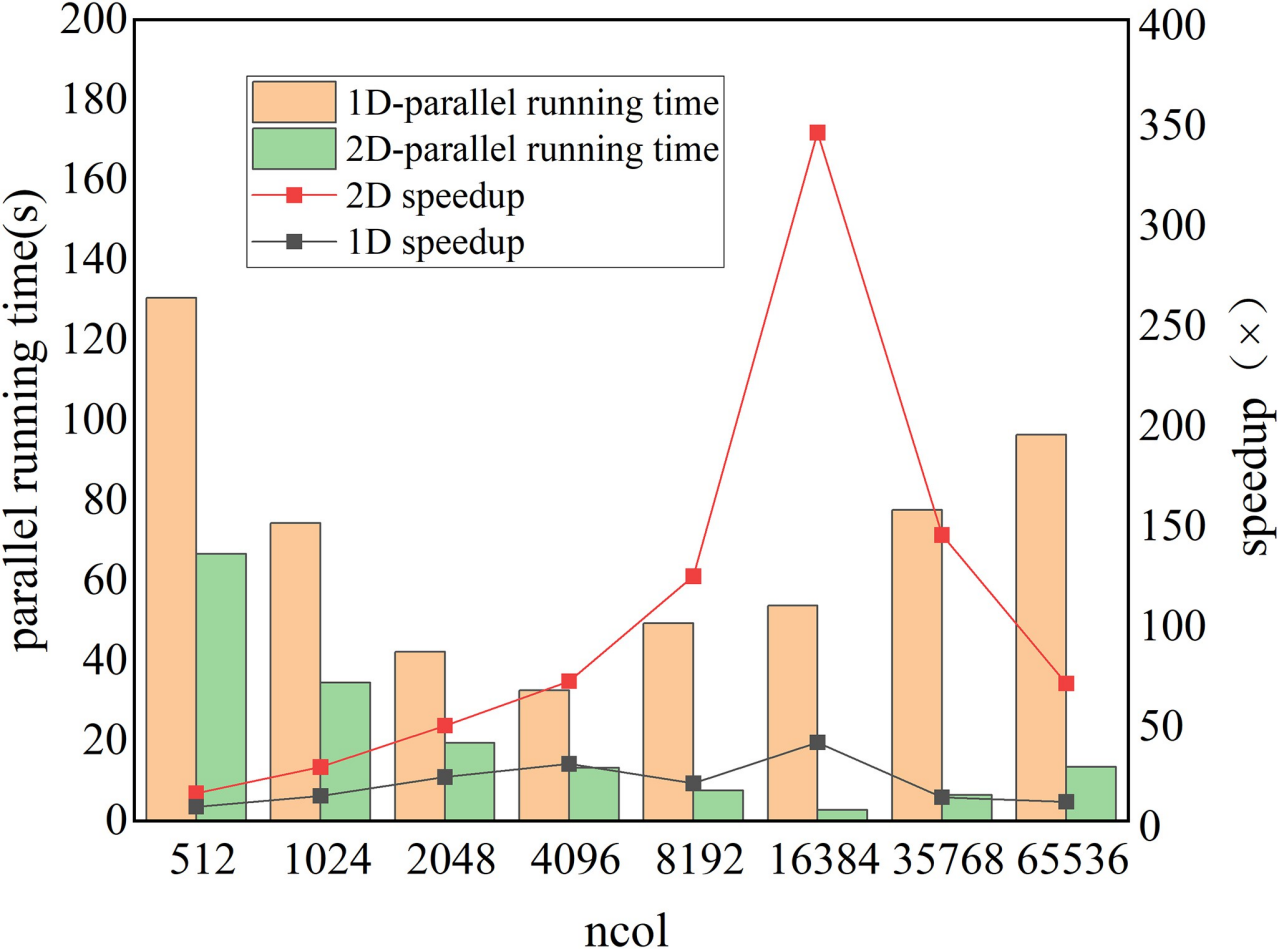
The impact of ncol on the runtime (s) and speedup of the ZM algorithm on a single A100 GPU, where the block size is 256.

ZM runtime is 927.8s on a single Intel Xeon E5-2680 v2 CPU. Fig 8 shows the runtime of a GPU-ZM with parallel acceleration on a single CPU and optimal ncol without considering data transfer. Where the bar represents the running time of the function, and the line is the speedup of the function.

**Fig 9:**
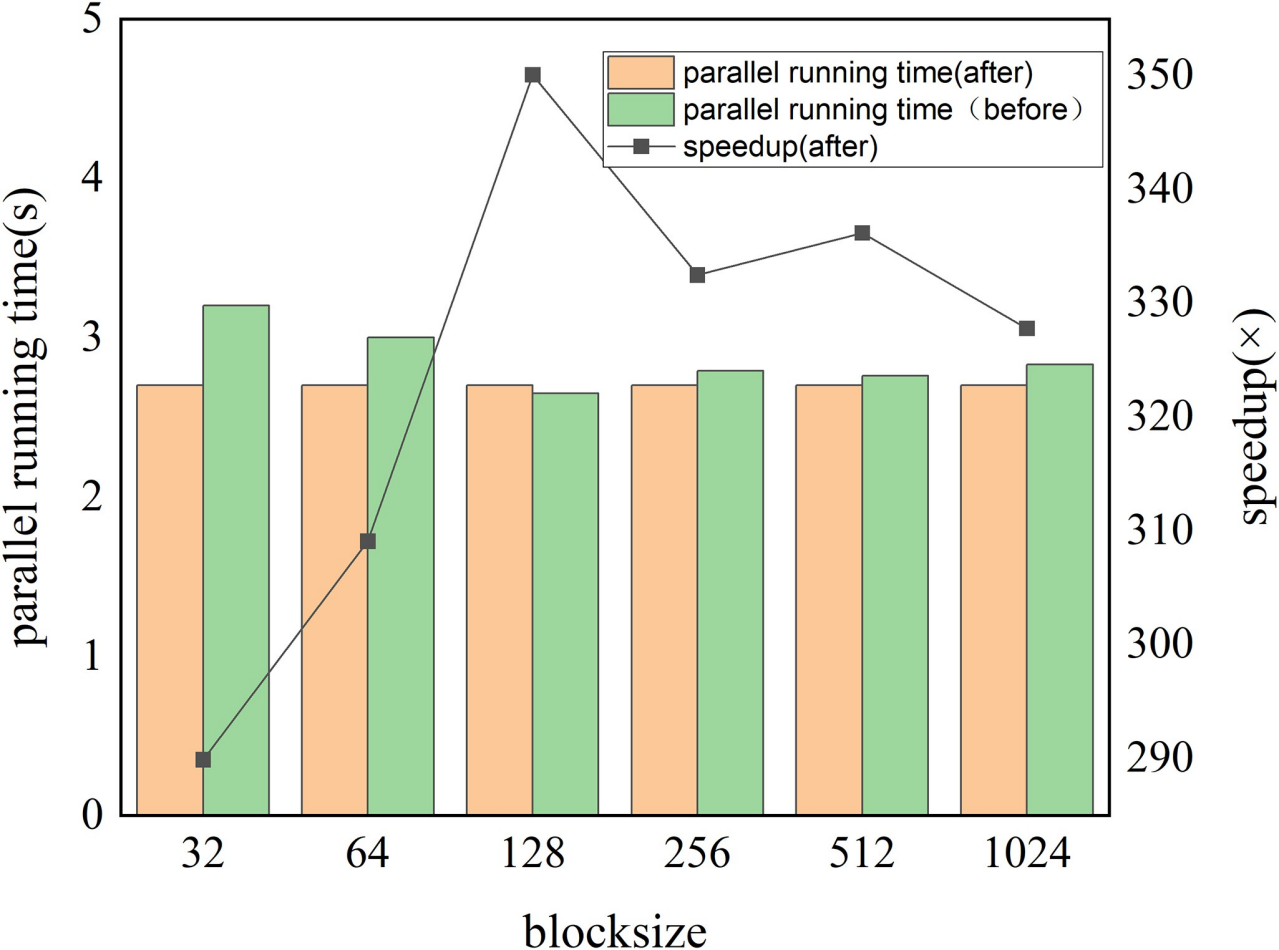
The influence of block size on the runtime (s) of ZM on one A100 GPU, where the ncol = 65536.

We investigate the optimal configuration of the thread block shape and evaluate the impact of varying thread block sizes on the program’s performance. and in comparison, we find that when the thread block organization is 128, the function can obtain the optimal computational efficiency, which is more efficient on the basis of experiment 1, reaching 366.72×, and the specific acceleration effect is shown in Fig 9, without I/O transmission.

### 5.5 Effect of CUDA streams

In this experimental analysis section, we can further expand on the effects of using CUDA streaming technology. First, we can discuss in detail the impact of CUDA streaming technology on data transfer time.

According to the experimental results, the data transmission time is significantly reduced after using CUDA streaming technology. This is because CUDA streaming technology can perform data transfer in parallel with device computing tasks, improving overall efficiency. By decoupling data transfer from computing tasks and executing them on parallel processors, you can make full use of the computing resources of the device while reducing the wait time for data transfer.

**Fig 10:**
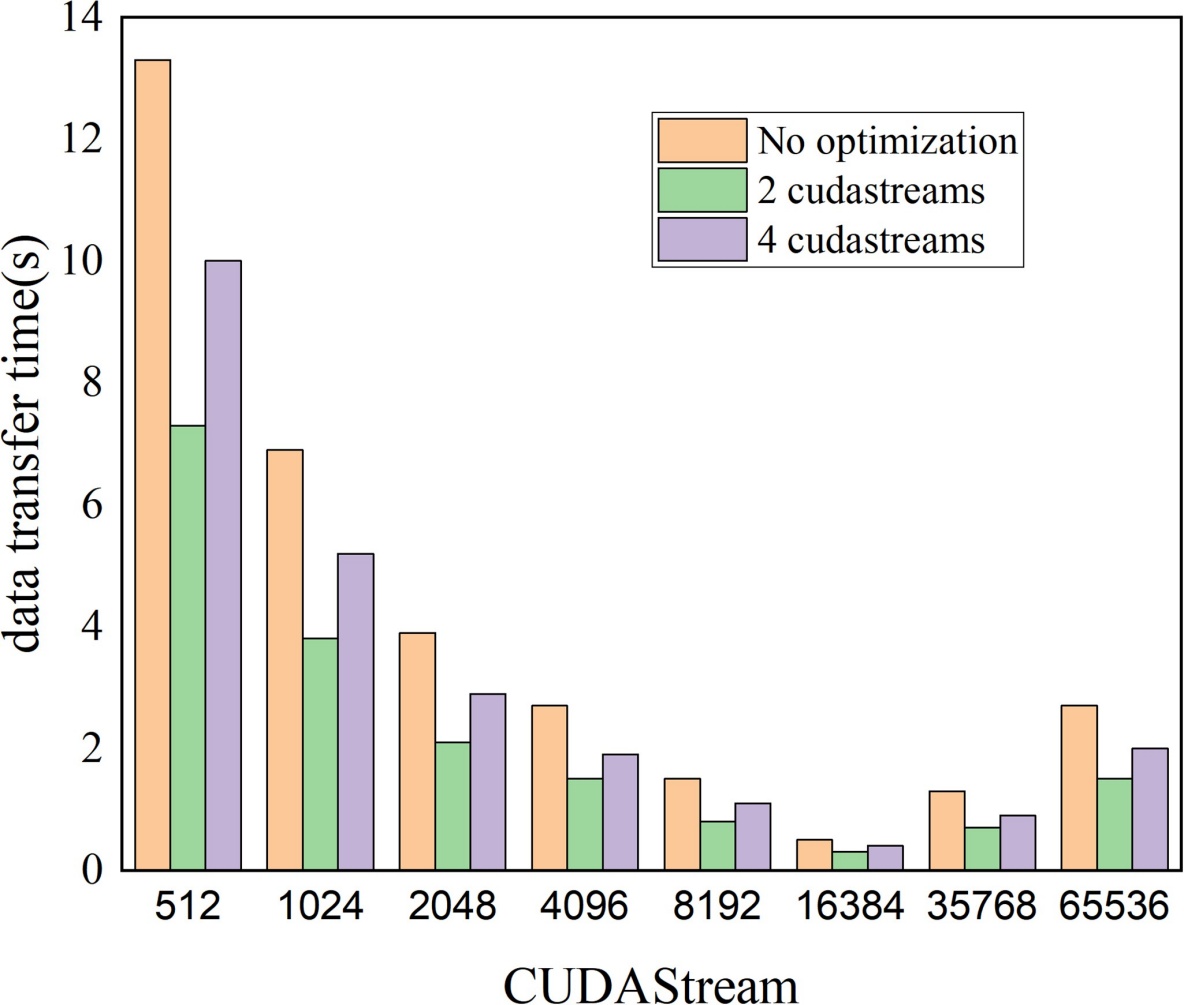
The data transfer time in different ncol cases when using CUDA stream technology.

However, when the number of CUDA streams increases to 4, the kernel function synchronization time also increases as shown in Fig 10, Tables 2 and 3. This is because when multiple CUDA streams are executed at the same time, there needs to be synchronization between the kernel functions to ensure the correctness of the concurrent execution. Synchronization operations introduce a certain amount of overhead, which increases the synchronization time of the kernel functions.

**Table 2:** Data transfer time (s) from CPU to GPU in different ncol cases when using CUDA stream technology.

ncol	no optimization	2 CUDA streams	4 CUDA streams
512	7.73	4.82	3.93
1024	3.62	2.63	3.89
2048	2.21	1.43	2.04
4096	1.86	1.02	1.29
8192	1.01	0.56	0.77
16384	0.34	0.23	0.32
35768	0.85	0.53	0.63
65536	1.85	1.01	1.83

**Table 3:** Data transfer time (s) from GPU to CPU in different ncol cases when using CUDA stream technology.

ncol	no optimization	2 CUDA streams	4 CUDA streams
512	6.21	2.41	6.33
1024	3.31	1.22	1.74
2048	1.63	0.73	0.91
4096	0.93	0.52	0.63
8192	0.52	0.27	0.36
16384	0.16	0.11	0.11
35768	0.41	0.25	0.33
65536	0.91	0.52	0.62

To better understand this phenomenon, we can further explore the sources of synchronous time. First of all, synchronization can be due to data dependencies. When there is a data dependency between multiple kernel functions, subsequent kernel functions need to wait for the previous kernel functions to complete the computation and data transmission to ensure data consistency. This wait leads to an increase in the synchronization time.

In addition, the synchronization time can be affected by the limitations of the device’s resources. When compute resources on a device are limited, the concurrent execution of multiple core functions can cause resource contention, increasing synchronization time. In addition, factors such as memory, bandwidth, and cache in the device can also be bottlenecks in synchronization time.

## 6 Conclusions and future work

Designing an efficient GPU-based acceleration algorithm for deep convective physical parameterization schemes is a huge challenge. This paper proposed a 2D acceleration algorithm for the ZM scheme on GPU. On A100 GPU, the ZM has a speedup of 366.72×. Moreover, after the performance optimization method for data transmission between CPU and GPU, the transmission time is 1.6× faster than before. The results show that the proposed algorithms and optimization method are effective.

The future work is mainly in two aspects: (1) The deep convection physical parameterization scheme is currently only implemented on a single GPU, rather than on multiple GPUs, and in order to make full use of the thousands of CPU and GPU nodes in the GPU cluster, hybrid programming mode MPI+CUDA should be considered to achieve accelerate on multiple GPUs [21]. Obviously, integrating the implementation of the program on multiple GPUs can be a huge challenge. But at the same time, algorithms based on multiple GPUs will get better acceleration. (2) We plan to further enhance the efficiency of deep learning algorithms by introducing mixed-precision computing. By converting model parameters and computational graph operations to half-precision floating-point numbers, we can reduce memory consumption and bandwidth requirements, thereby improving computational speed. Our future research will involve experimental investigations to optimize the parameters and techniques of mixed-precision computing, while also considering its impact on model stability and accuracy. We believe that mixed-precision computing will become a crucial strategy for optimizing deep learning algorithms, facilitating breakthroughs in various domains.

## Supporting information

S1 File (CU)

## References

[1] Zhang Y, Yang B, Liu Y, et al. High-precision Ecological Protection Red Line Boundary Optimization for Fangshan District, Beijing, China. Sensors & Materials, 2023, 35.

[2] Xiao D, Tong-Hua S, Jun W, et al. Decadal variation of the Aleutian Low-Icelandic Low seesaw simulated by a climate system model (CAS-ESM-C). Atmospheric and Oceanic Science Letters, 2014, 7(2): 110–114. doi: 10.1080/16742834.2014.11447144

[3] Zhang H, Zhang M, Zeng Q. Sensitivity of simulated climate to two atmospheric models: Interpretation of differences between dry models and moist models. Monthly Weather Review, 2013, 141(5): 1558–1576. doi: 10.1175/MWR-D-11-00367.1

[4] Fan Z, Qiu F, Kaufman A, et al. GPU cluster for high performance computing. SC’04: Proceedings of the 2004 ACM/IEEE conference on Supercomputing. IEEE, 2004: 47–47.

[5] Deng Z, Chen D, Hu Y, et al. Massively parallel non-stationary EEG data processing on GPGPU platforms with Morlet continuous wavelet transform. Journal of Internet Services and Applications, 2012, 3: 347–357. doi: 10.1007/s13174-012-0071-1

[6] Morrison H, Curry J A, Khvorostyanov V I. A new double-moment microphysics parameterization for application in cloud and climate models. Part I: Description. Journal of the Atmospheric Sciences, 2005, 62(6): 1665–1677. doi: 10.1175/JAS3446.1

[7] Chen D, Wang L, Tian M, et al. Massively parallel modelling & simulation of large crowd with GPGPU. The Journal of Supercomputing, 2013, 63: 675–690. doi: 10.1007/s11227-011-0675-4

[8] Hayes A B, Hua F, Huang J, et al. Decoding CUDA binary. 2019 IEEE/ACM International Symposium on Code Generation and Optimization (CGO). IEEE, 2019: 229–241.

[9] Owens J D, Houston M, Luebke D, et al. GPU computing. Proceedings of the IEEE, 2008, 96(5): 879–899. doi: 10.1109/JPROC.2008.917757

[10] Mielikainen J, Huang B, Huang H L A, et al. Improved GPU/CUDA based parallel weather and research forecast (WRF) single moment 5-class (WSM5) cloud microphysics. IEEE Journal of Selected Topics in Applied Earth Observations and Remote Sensing, 2012, 5(4): 1256–1265. doi: 10.1109/JSTARS.2012.2188780

[11] Mielikainen J, Huang B, Wang J, et al. Compute Unified Device Architecture (CUDA)-based parallelization of WRF Kessler cloud microphysics scheme. Computers & Geosciences, 2013, 52: 292–299. doi: 10.1016/j.cageo.2012.10.006

[12] Mielikainen J, Huang B, Huang H L A, et al. Speeding up the computation of WRF double-moment 6-class microphysics scheme with GPU. Journal of Atmospheric and Oceanic Technology, 2013, 30(12): 2896–2906. doi: 10.1175/JTECH-D-12-00218.1

[13] Leutwyler D, Fuhrer O, Lapillonne X, et al. Towards European-scale convection-resolving climate simulations with GPUs: A study with COSMO 4.19. Geoscientific Model Development, 2016, 9(9): 3393–3412. doi: 10.5194/gmd-9-3393-2016

[14] Cao H, Yuan L, Zhang H, et al. AGCM-3DLF: Accelerating atmospheric general circulation model via 3-D parallelization and leap-format[J]. IEEE Transactions on Parallel and Distributed Systems, 2022, 34(3): 766–780. doi: 10.1109/TPDS.2022.3231013

[15] Huang M, Huang B, Gu L, et al. Parallel GPU architecture framework for the WRF Single Moment 6-class microphysics scheme. Computers & Geosciences, 2015, 83: 17–26. doi: 10.1016/j.cageo.2015.06.014

[16] Kim J Y, Kang J S, Joh M. GPU acceleration of MPAS microphysics WSM6 using OpenACC directives: Performance and verification. Computers & Geosciences, 2021, 146: 104627. doi: 10.1016/j.cageo.2020.104627

[17] Wang Z, Wang Y, Wang X, et al. GPU-RRTMG_SW: Accelerating a shortwave radiative transfer scheme on GPU. IEEE Access, 2021, 9: 84231–84240. doi: 10.1109/ACCESS.2021.3087507

[18] Li F, Wang Y, Wang Z, et al. CC-RRTMG_SW++: Further optimizing a shortwave radiative transfer scheme on GPU. The Journal of Supercomputing, 2022, 78(15): 17378–17402. doi: 10.1007/s11227-022-04566-5

[19] Hong Y, Wang Y, Zhang X, et al. A GPU-enabled acceleration algorithm for the CAM5 cloud microphysics scheme. The Journal of Supercomputing, 2023, 79(16): 17784–17809. doi: 10.1007/s11227-023-05360-7

[20] Zhang G J. Convective quasi‐equilibrium in midlatitude continental environment and its effect on convective parameterization. Journal of Geophysical Research: Atmospheres, 2002, 107(D14): ACL 12-1–ACL 12-16. doi: 10.1029/2001JD001005

[21] Wang Y, Zhao Y, Jiang J, et al. A novel GPU-based acceleration algorithm for a longwave radiative transfer model. Applied Sciences, 2020, 10(2): 649. doi: 10.3390/app10020649

